# Parathyroid Storm Secondary to a Parathyroid Adenoma Presenting With Recurrent Generalised Tonic-Clonic Seizures and Refractory Hypercalcaemia: A Case Report

**DOI:** 10.7759/cureus.116719

**Published:** 2026-09-22

**Authors:** Reem Abdel-Aal, Mahzaib Babar, Keshav Harivardhan, Isuri Kurera, Jennifer Tringham

**Affiliations:** 1 Internal Medicine, Frimley Park Hospital, Farnborough, GBR; 2 Endocrinology and Diabetes, Frimley Park Hospital, Farnborough, GBR; 3 Diabetes and Endocrinology, Frimley Park Hospital, Farnborough, GBR

**Keywords:** hyperparathyroidism treatment, metabolic hypercalcaemia, parathyroidectomy, parathyroid storm, urgent parathyroidectomy

## Abstract

Parathyroid storm is a rare, life-threatening manifestation of primary hyperparathyroidism characterised by severe hypercalcaemia and acute multisystem deterioration. We report a 70-year-old woman with known primary hyperparathyroidism who presented with progressive weakness, confusion and reduced mobility and was found to have a corrected serum calcium concentration of 4.14 mmol/L. Despite aggressive intravenous hydration, she developed recurrent generalised tonic-clonic seizures, hypoventilation and reduced consciousness, requiring intubation and intensive care admission. Electroencephalography demonstrated severe diffuse cerebral dysfunction consistent with metabolic encephalopathy, while cerebrospinal fluid studies were unremarkable. Four-dimensional computed tomography of the neck identified a 1.8 cm right-sided parathyroid lesion. Hypercalcaemia remained difficult to control despite multimodal medical therapy, including pamidronate, calcitonin, cinacalcet and denosumab, with recurrent rebound hypercalcaemia. Urgent parathyroidectomy resulted in sustained normalisation of serum calcium and cessation of seizure activity, and histopathology confirmed a benign parathyroid adenoma. This case highlights the severe neurological complications of parathyroid storm and the importance of early multidisciplinary consideration of definitive surgery when hypercalcaemia remains refractory to intensive medical therapy.

## Introduction

Parathyroid storm, also termed hyperparathyroid or hypercalcaemic crisis, is a rare but potentially life-threatening manifestation of primary hyperparathyroidism (PHPT), characterised by severe symptomatic hypercalcaemia with acute multisystem deterioration [1-3]. Unlike uncomplicated hypercalcaemia, it is characterised by marked calcium elevation accompanied by acute clinical deterioration and multiorgan involvement. Neurological manifestations range from weakness, confusion and altered consciousness to severe encephalopathy; seizures are uncommon but have been reported in association with profound hypercalcaemia secondary to PHPT [2,4]. Prompt recognition, aggressive medical stabilisation and timely consideration of definitive parathyroid surgery are essential [2,3,5]. We report an unusual presentation of parathyroid storm with recurrent generalised tonic-clonic seizures and severe hypercalcaemia refractory to intensive medical therapy, ultimately requiring urgent parathyroidectomy.

## Case presentation

A 70-year-old woman with a background history of known PHPT was first identified approximately 23 months before the index admission, when routine blood tests in primary care showed a corrected calcium of 2.81 mmol/L and parathyroid hormone (PTH) of 17.5 pmol/L. She was subsequently referred to endocrinology. At her first endocrinology appointment, approximately three months before admission, her calcium had increased to 2.97 mmol/L with a PTH of 34.8 pmol/L, and she reported symptoms of hypercalcaemia, including increased urination. She was started on cinacalcet 30 mg once daily, with an initial improvement in calcium to 2.72 mmol/L approximately two months before admission. She was also found to have vitamin D deficiency (13.8 nmol/L) and was started on vitamin D replacement. Despite treatment, her calcium remained elevated at 2.82 mmol/L approximately one month before admission. A bone density scan also showed osteoporosis, and alendronic acid was commenced.

On Day 0, she was admitted with severe hypercalcaemia, with a corrected calcium of 4.14 mmol/L. She developed a generalised tonic-clonic seizure on the day of admission, with a further seizure occurring during her subsequent clinical course. This was followed by hypoventilation and reduced consciousness, necessitating intubation and admission to the intensive care unit. She received 8 litres of 0.9% intravenous sodium chloride during the first 48 hours while treatment of the hypercalcaemic crisis continued. Further investigations excluded alternative neurological and infective causes of the seizures and encephalopathy.

Investigations

Initial biochemical investigations revealed severe hypercalcaemia, with a corrected serum calcium of 4.14 mmol/L and PTH of 112.7 pmol/L, in the context of known PHPT. Previous outpatient investigations had shown persistently elevated PTH concentrations (34.8 and 45.4 pmol/L), vitamin D deficiency, and a low 24-hour urinary calcium-to-creatinine clearance ratio. This is demonstrated in Table 1, which highlights the key investigations undertaken during admission.

**Table 1:** Summary of key investigations during admission. *PTH reference intervals are assay dependent; the stated adult interval is used here for context and reflects the local laboratory reference range.

Investigation	Result	Reference value	Interpretation
Adjusted calcium (Day 0)	4.14 mmol/L	2.20-2.60 mmol/L	Hypercalcaemic crisis
Parathyroid hormone (PTH) (Day 0)	112.7 pmol/L	2.30-14.0 pmol/L*	Inappropriately elevated
Dual-energy X-ray absorptiometry (DEXA)	Osteoporosis	Not applicable	End-organ manifestation
Electroencephalography (EEG)	Diffuse encephalopathy	Not applicable	Metabolic encephalopathy
Lumbar puncture	Negative	Not applicable	No central nervous system infection
Four-dimensional computed tomography (4D CT)	1.8 cm right-sided lesion	Not applicable	Parathyroid adenoma
Histopathology	Right parathyroid adenoma	Not applicable	No malignant features

Electroencephalography showed severe diffuse cerebral dysfunction consistent with metabolic encephalopathy. Cerebrospinal fluid analysis was unremarkable, with negative microbiological studies, making central nervous system infection unlikely.

Although sestamibi scintigraphy and parathyroid ultrasonography are commonly used as first-line localisation studies, 4D CT was chosen in this case because of its higher diagnostic yield and the practical advantage of being readily performed during the inpatient admission. Four-dimensional computed tomography of the neck identified a 1.8 cm heterogeneously enhancing right-sided parathyroid lesion, which is highlighted in Figure 1. Following parathyroidectomy, histopathological examination confirmed a benign parathyroid adenoma without malignant features. The biochemical findings, radiological localisation of the lesion, and postoperative normalisation of serum calcium supported the diagnosis of a functioning parathyroid adenoma causing PHPT [1].

**Figure 1:**
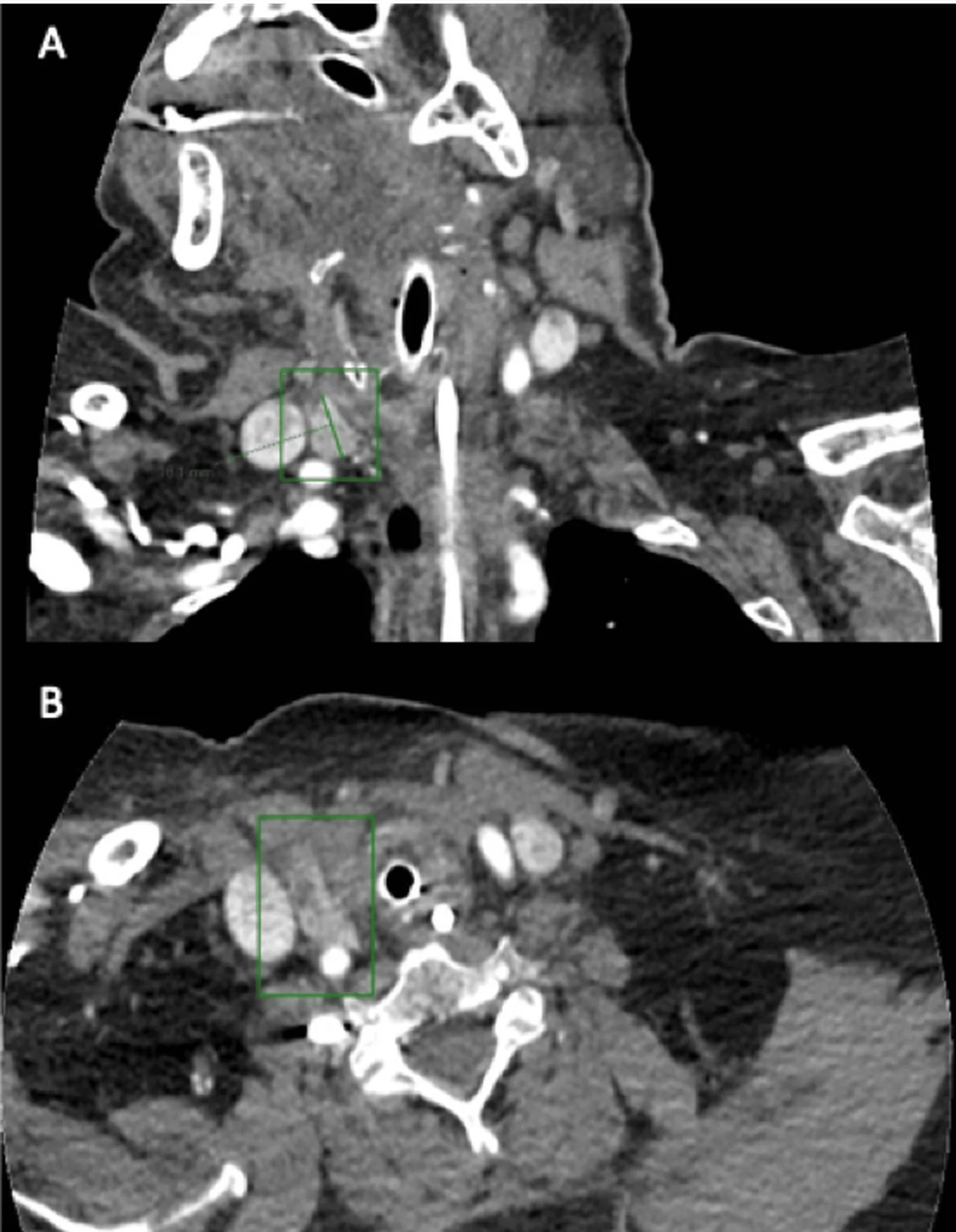
Four-dimensional computed tomography (4D CT) of the neck demonstrating a 1.8 cm right parathyroid lesion, radiologically consistent with a parathyroid adenoma: (A) coronal image and (B) axial image. The lesion in the right parathyroid region is indicated by the green annotation.

Differential diagnoses

Differential diagnoses for the patient's seizures and encephalopathy included metabolic encephalopathy secondary to severe hypercalcaemia, viral encephalopathy, cerebrovascular disease and a primary seizure disorder. Severe hypercalcaemia is recognised to cause neurological dysfunction, including confusion, impaired consciousness and, rarely, generalised tonic-clonic seizures [2,4]. Normal cerebrospinal fluid studies made an infective cause unlikely, while electroencephalography supported a diffuse metabolic cerebral process. The overall biochemical, radiological and postoperative findings were consistent with severe PHPT secondary to a parathyroid adenoma.

Treatment

The patient received 8 L of intravenous 0.9% sodium chloride during the first 48 hours, alongside calcium-lowering therapy with intravenous pamidronate 60 mg (repeated doses, with a subsequent 30 mg dose), salmon calcitonin 100 units three times daily, escalating doses of cinacalcet up to 60 mg twice daily, denosumab (90 mg followed by two 60 mg doses), and prednisolone 30 mg once daily for three days. Cinacalcet can reduce serum calcium in PHPT, while multimodal calcium-lowering therapy may be required in severe hypercalcaemic crisis [1,6]. The timing of major clinical events and calcium-lowering interventions is summarised in Table 2.

**Table 2:** Summary of key clinical events during admission including medical interventions (intravenous fluids, pamidronate, calcitonin, denosumab and parathyroidectomy).

Day	Clinical event	Category
Day 0	First seizure	Neurological event
Day 1	Pamidronate administered	Medical management
Days 2-3	Calcitonin 100 international units three times daily	Medical management
Day 4	Second seizure	Neurological event
Day 21	Pamidronate administered	Medical management
Days 30-34	Calcitonin 100 international units three times daily	Medical management
Day 39	Pamidronate 30 mg administered	Medical management
Day 40	Denosumab 120 mg subcutaneously administered	Medical management
Day 44	Parathyroidectomy	Definitive management

Despite an initial improvement in adjusted calcium from 4.14 mmol/L to 2.65 mmol/L, the patient remained significantly deconditioned following her ICU admission, with persistent delirium and very limited mobility. During this period, her calcium began to rise rapidly again, reaching 3.74 mmol/L despite repeated and aggressive medical therapy. Similar difficulty in achieving sustained biochemical control despite combinations of intravenous fluids, calcitonin, antiresorptive therapy and cinacalcet has been described in severe hyperparathyroid crisis [2,6].

Her case was discussed repeatedly within the multidisciplinary team, with input from endocrinology, endocrine surgery, anaesthetics and intensive care. Given her poor functional and neurological state, she was considered to be at high risk for surgery and general anaesthesia. However, as her calcium continued to rise despite medical treatment and there was no sustained biochemical or neurological improvement, urgent parathyroidectomy was felt to be the only definitive treatment. The decision was therefore made to proceed with surgery despite the significant perioperative risk. As the patient remained delirious and lacked capacity to consent, the decision to proceed was made in her best interests following discussion with the multidisciplinary team and her family.

Outcome and follow-up

Serum calcium rapidly normalised following parathyroidectomy and remained within the reference range thereafter (Figure 2). The postoperative course was complicated by a neck haematoma requiring surgical evacuation and drain insertion. Histopathological examination confirmed a benign parathyroid adenoma with no malignant features.

**Figure 2:**
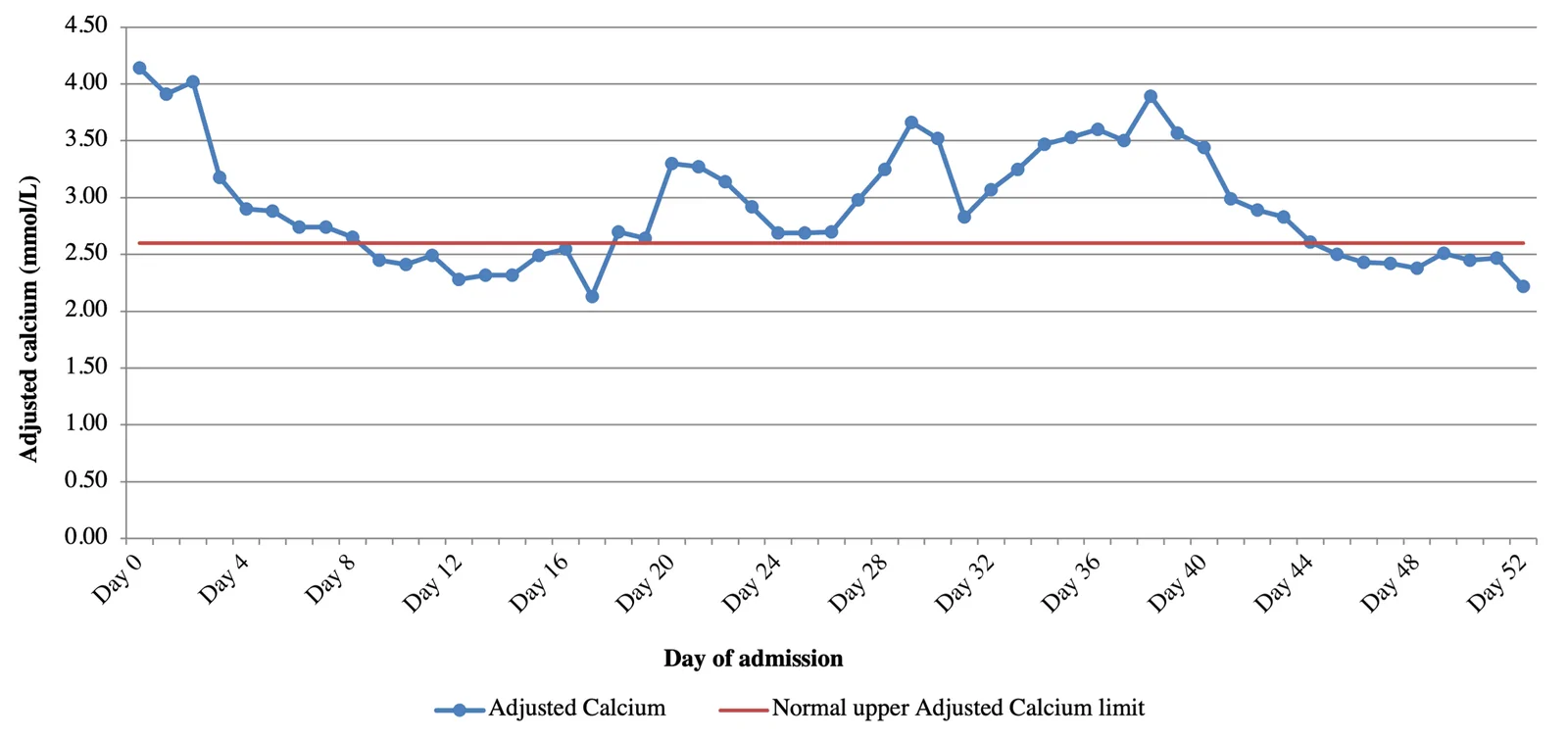
Serial adjusted serum calcium concentrations during admission, displayed using Day 0 as the index admission date. Repeated medical therapies produced transient biochemical responses, whereas parathyroidectomy on Day 44 was followed by sustained normalisation.

The patient subsequently made a progressive neurological recovery with no further seizures. Following physiotherapy and rehabilitation, she was discharged home mobilising independently with a walking stick.

## Discussion

Parathyroid storm is an uncommon but potentially fatal manifestation of PHPT. It is characterised by severe hypercalcaemia accompanied by rapid systemic deterioration, with neurological, renal, gastrointestinal and cardiovascular manifestations described [2,7]. Although confusion, lethargy and altered consciousness are established neurological consequences of severe hypercalcaemia, generalised tonic-clonic seizures are considerably less common [2,4]. Published reports of PHPT associated with severe hypercalcaemia have nevertheless demonstrated that seizures and marked alterations in consciousness can occur as part of the neurological presentation [4].

The initial management of severe symptomatic hypercalcaemia requires rapid intravenous volume expansion, followed where appropriate by pharmacological calcium-lowering therapies such as intravenous bisphosphonates and calcitonin [3]. In PHPT, cinacalcet may reduce serum calcium concentrations, particularly when surgery cannot immediately be undertaken [1]. However, medical treatment in hyperparathyroid crisis should principally provide physiological stabilisation while definitive treatment is arranged rather than replace definitive surgery [2,5-10].

This case particularly demonstrates the limitations of medical management in severe PTH-mediated hypercalcaemia. Despite intravenous hydration and multiple calcium-lowering agents, the patient's calcium remained difficult to control, with recurrent rebound hypercalcaemia occurring in association with profound neurological complications. A similarly severe case reported by Gill et al. demonstrated persistent hypercalcaemia despite fluid resuscitation, denosumab, calcitonin, pamidronate and cinacalcet, ultimately requiring urgent parathyroidectomy [6].

Evidence from larger case series and systematic reviews supports an early surgical strategy once patients with hyperparathyroid crisis have been sufficiently stabilised [2,7-10]. Gurrado et al., in a systematic review of reported hypercalcaemic crises secondary to PHPT, identified parathyroid adenoma as the most frequent underlying histological diagnosis and concluded that medical therapy followed by expeditious parathyroidectomy should be regarded as the treatment of choice [7]. Long-term surgical series have likewise reported durable biochemical control following parathyroidectomy in patients presenting with hypercalcaemic crisis [8,10].

Early multidisciplinary collaboration in the present case facilitated localisation of the adenoma, intensive medical stabilisation and timely parathyroidectomy. Definitive removal of the adenoma resulted in sustained biochemical remission accompanied by cessation of seizure activity and progressive neurological recovery.

Finally, this case demonstrates that benign histology does not predict the functional severity of parathyroid disease. A histologically benign parathyroid adenoma can generate profound PTH-mediated hypercalcaemia and precipitate a life-threatening endocrine emergency [1,6-10].

Learning outcomes

This case highlights the importance of considering timely parathyroidectomy in severe hypercalcaemic crisis at the earliest opportunity once the patient is clinically stable for anaesthesia. Clinicians should anticipate post-parathyroidectomy hypocalcaemia and ensure close biochemical monitoring in an HDU setting. Severe hypercalcaemia should be recognised as a potential cause of seizures and metabolic encephalopathy, requiring prompt calcium-lowering therapy and multidisciplinary management. Furthermore, initial improvement with medical therapy may be transient, and recurrent or refractory hypercalcaemia should prompt early escalation to definitive surgical treatment.

## Conclusions

Parathyroid storm is a rare but potentially life-threatening endocrine emergency in which profound hypercalcaemia can produce severe neurological manifestations, including generalised tonic-clonic seizures and metabolic encephalopathy. Initial management requires rapid correction of volume depletion and intensive calcium-lowering therapy, but persistent or recurrent hypercalcaemia despite multimodal medical treatment should prompt early multidisciplinary consideration of definitive parathyroidectomy. In this case, removal of a histologically benign parathyroid adenoma resulted in sustained biochemical normalisation, cessation of seizure activity and progressive neurological recovery, illustrating both the limitations of medical therapy in refractory parathyroid crisis and the fact that benign histology does not preclude life-threatening functional disease.
